# An *in vitro* study of neuroprotective properties of traditional Chinese herbal medicines thought to promote healthy ageing and longevity

**DOI:** 10.1186/1472-6882-13-373

**Published:** 2013-12-27

**Authors:** Bojiang Shen, John Truong, Ray Helliwell, Suresh Govindaraghavan, Nikolaus J Sucher

**Affiliations:** 1 Centre of Complementary Medicine Research, School of Science and Health, University of Western Sydney, Locked Bag 1797, Penrith, NSW 2751, Australia; 2 Lipa Pharmaceuticals, Sydney, Australia; 3 Network Nutrition-IMCD Australia, Unit 9, 7 Meridian Place, Bellow Vista, NSW 2153, Australia; 4 STEM Division, Roxbury Community College, 1234 Columbus Avenue, Roxbury Crossing, MA 02120, USA

**Keywords:** Neuroprotection, Chinese herbal medicine, Herbal extract, Apoptosis, Resveratrol

## Abstract

**Background:**

Age is the leading risk factor for acute and chronic neurodegenerative diseases. The Shen Nong Ben Cao Jing, the oldest known compendium of Chinese materia media, lists herbal medicines that were believed to exert neither fast acting pharmacological effects nor discernible toxicity, but to promote general health and longevity. In modern terms, these herbal medicines could be considered as complementary health care products for prevention rather than treatment of diseases. In the present study, we examined whether a selection of 13 such herbal medicines exhibited neuroprotective activity.

**Methods:**

The antioxidant capacity of the herbal extracts was determined using three non-cellular assays measuring the total phenol content (FCR assay), 2,2-diphenyl-1-picrylhydrazyl (DPPH) radical scavenging capacity and oxygen radical absorbance capacity (ORAC). Cytotoxic effects of the herbal extracts were assayed in cultured mouse cortical neurons and their neuroprotective activities were studied using staurosporine-induced apoptosis of the cultured neurons.

**Results:**

Most of the herbal extracts showed negligible toxic effects at 100 μg/ml. However*, Polygonum multiflorum* and *Rhodiola rosea* exhibited some neurotoxicity at this concentration. Extracts of *Ganoderma lucidum*, *Glycyrrhiza glabra*, *Schizandra chinensis*, and *Polygonum cuspidatum* inhibited staurosporine-induced apoptosis by 30 – 50% in a dose-dependent manner. The neuroprotective effects of *Polygonum cuspidatum* were predominantly due to its major ingredient, resveratrol. The effective herbal extracts showed various levels of reactive oxygen species (ROS) scavenging capacity, which was significantly correlated with their neuro- protective activity. However, *P. multiflorum* and *R. rosea* extracts proved to be the exception as they exhibited a high level of antioxidant capacity, but did not exhibit neuroprotective effects in cell-based assay.

**Conclusions:**

This *in vitro* study provides evidence for neuroprotective activity of some Chinese herbal medicines traditionally used to promote healthy ageing and longevity. Our results provide a justification for further study of these herbal extracts in neurodegenerative animal models to assess their safety and effectiveness as a basis for subsequent clinical trials. These herbal medicines might potentially offer a novel preemptive neuroprotective approach in neurodegenerative diseases and might be developed for use in persons at risk.

## Background

Age is the leading risk factor for acute and chronic neurodegenerative diseases such as stroke, Parkinson’s and Alzheimer’s disease. As population ageing is occurring on a global scale, the incidence of these diseases is likely to increase significantly in the near future. To date, there is a lack of effective preventive strategies for these disorders. Furthermore, treatments are mainly symptomatic and can at best temporarily slow down disease progression. A large body of basic molecular neuroscience research over the last 30 years has led to the elucidation of the molecular mechanisms involved in the events leading to neuronal injury and death. Multiple lines of evidence from a large number of *in vitro*, *in vivo* and post mortem studies indicate that the demise of neurons is often the result of the activation of common (shared) cell death programs such as apoptosis [1]. Research has also demonstrated that pharmacological or genetic manipulation of the underlying molecular pathways can protect neurons from deadly insults [2-4]. The strategy aimed at antagonizing, interrupting or slowing the molecular events leading to irreversible injury or death of neurons in neurodegenerative diseases is commonly referred to as neuroprotection [5-7]. However, the poor translation from animal studies to clinical trials has disappointed neurologists and aroused hot debate on reasons for the apparent failure of neuroprotection as an effective treatment for neurodegenerative diseases [7-10].

A lack of treatment options has led to an increasing number of people to use “natural” and herbal medicines in an attempt to prevent or delay the deleterious effects of ageing. Longevity and good health have always been desirable goals for humans. The Shen Nong Ben Cao Jing (Shen Nong’s Herbal; compiled some 2000 years ago) is the first known example of an extensive body of literature devoted to the description and classification of the Chinese materia medica and many later books on Chinese materia medica carry the term “ben cao” in their title. It contains the description of some 365 drugs that were grouped into three classes. The drugs in the top class neither exerted fast acting pharmacological effects nor any discernible toxicity. They were believed to promote general health and longevity when they were taken daily on a long-term basis. In modern terms, these drugs could be considered as complementary health care products such as functional foods or nutraceuticals aimed at promoting health and the prevention rather than the treatment of diseases.

Oxidative stress has been implicated in the pathogenesis of both acute and chronic neurodegenerative diseases and is believed to play a role in ageing, which is the key risk factor for neurodegenerative diseases [11]. Chinese herbal medicines are known to be a rich source of antioxidants and their purported therapeutic effects are often linked to their antioxidant activity [12]. Along these lines, we set out to investigate whether or not a sample of Chinese herbal medicines used to promote general health and longevity exhibit cytoprotective effects towards neurons and glia in the central nervous system and to test the hypothesis that any such effects were correlated with their antioxidant activity. Here we report that some of the selected herbal extracts exhibited neuroprotective activity in a well-established in vitro model of neuronal apoptosis induced by the non-selective protein kinase inhibitor staurosporine [13,14]. While the neuroprotective activity was correlated with the antioxidant capacity of the extracts, two extracts (*Polygonum multiflorum* and *Rhodiola rosea*) with high anti-oxidant capacity lacked neuroprotective activity in the staurosporine assay and exhibited some neurotoxic activity when they were applied in the absence of staurosporine. These results complement our previous study reporting on the cytoprotective properties of these extracts in glial cells [15]. Interestingly, some extracts exhibited selective protection in neurons but not glia (*Polygonum cuspidatum*) or glia but less so in neurons (*P. multiflorum*). Together, the data appear to support the traditional practice of combining multiple herbal medicines for the treatment of complex disorders [15]. The results of this study provide a justification for further testing of these herbal extracts in neurodegenerative animal models to assess their safety and effectiveness *in vivo* as a basis for subsequent clinical studies in humans. In the absence of known adverse effects, these herbal medicines might offer a novel preemptive neuroprotective approach in acute and chronic neurodegenerative diseases and might be developed for prophylactic use in persons at risk.

## Methods

### Herbal extracts and reagents

Commercially available extracts of medicinal herbs for use as listed herbal medicines, were provided by LIPA Pharmaceuticals Ltd (Minto, NSW, Australia). Traceability of the extracts to their respective (qualified botanist) authenticated starting medicinal herbs was established by the Quality Control Department at LIPA Pharmaceuticals. The herb names, extraction solvent(s) and ratios are listed in Additional file 1: Table S1. Among the herbal extracts, the *Polygonum cuspidatum* extract was standardized to contain 50% of *trans-*resveratrol. The root powder of *Radix Glycyrrhizae* was used without initial extraction. For preparation of the stock, the herbal extract powders were further extracted with 80% of methanol under sonication for 30 min, twice, with a 15 min interval. After centrifugation, the supernatant was filtered and evaporated under vacuum to obtain a dried powder, which was referred to as herbal extract and used in the reported experiments. The higher strength of organic solvent (80% methanol) was expected to maximize the extraction of both moderately polar and polar constituents in the commercial extracts obtained from varying strengths of hydro-alcoholic mixtures.

For the biological experiments, the dried herbal extracts were dissolved in dimethyl sulfoxide (DMSO) to obtain 100 mg/ml stock solutions. Pure compounds, which are possible active ingredients in selective herbal extracts, such as *trans-*resveratrol, glabridin, and schisandrin, were purchased from Phytomarker (Tianjin, China). The compounds were freshly dissolved in DMSO to obtain 85 – 130 mg/ml stock solutions and then further diluted to working concentrations as indicated in the figures. The culture media and reagents were purchased from Life Technologies. All other reagents were purchased from Sigma unless otherwise stated.

### Neuronal cell culture

The Animal Care and Ethics Committee of the University of Western Sydney approved the use of animals in this study. BALB/c laboratory mice at day 18 of gestation were purchased from the Animal Resources Centre, Western Australia. Cerebral cortical neurons from mouse embryos were isolated and cultured as described previously [16] with some modifications. Briefly, the cortex tissue was dissected and then digested with 0.25% trypsin, followed by triturating in Hank's balanced salt solution (HBSS, without Ca^2+^ and Mg^2+^) supplemented with 5 mM 4-(2-hydroxyethyl)-1-piperazineethanesulfonic acid (HEPES). The cells were filtered through 70 μm meshes into 2 vol. of HBSS containing Ca^2+^ and Mg^2+^. After centrifugation at 1000 rpm for 5 min, the cell pellet was re-suspended in plating medium consisting of neurobasal medium supplemented with 0.5 mM GlutaMax, 25 μM glutamate, 2% B27 supplement and 1% Pen-Strep (Life Technology). The cells were seeded in poly-D-lysine (PDL)-coated 96-well or 24-well culture plates. After 2–3 days, half of the medium was replaced with fresh culture medium and twice weekly thereafter until the cultures were used in the experiments. The neuronal cell cultures were maintained at 37°C, 5% CO_2_ in 95% air for up to 10 days.

### Neurotoxicity assay

The widely accepted staurosporine induced apoptosis model used in this study was based on the previously established methods [17,18] with some modifications. In preliminary experiments, primary mouse cortical neuron cultures were exposed to various concentrations of staurosporine (10 to 800 nM) followed by assessment of apoptotic cell death to establish a dose–response curve. Staurosporine-induced cell death was dose-dependent with an EC_50_ of 130 nM (data not shown). A staurosporine concentration of 500 nM induced maximum cell death and was therefore used for all subsequent experiments.

The cells were exposed to herbal extracts (100 μg/ml), staurosporine (500 nM) or, simultaneously, to staurosporine and herbal extracts. Half of the culture media were replaced with new media containing double the final concentrations of staurosporine and/or the herbal extracts. The cells were incubated at 37°C, 5% CO_2_ for 24 h. Toxicity was determined by measuring the release of lactate dehydrogenase (LDH) into the culture medium using the LDH cytotoxicity detection kit (Roche Applied Science, USA). The percentage of cytotoxicity was calculated using the formula:

$$\mathrm{Cytotoxicity}\left(\%\right)=\left(A‒C_{\min}\right)/\left(C_{\max}‒C_{\min}\right)\times 100\%$$

A = absorbance measured; C_min_ = absorbance of negative control (medium contains vehicle only); C_max_ = absorbance of maximum toxicity (medium contains 500 nM staurosporine).

Non-cellular antioxidant capacity assays DPPH (2,2-diphenyl-1-picrylhydrazyl) radical scavenging assay, oxygen radical absorbance capacity (ORAC) assay and total phenol assay by Folin-Ciocalteau reagent were performed as described in detail previously [15].

### LC-ESI-MS analyses

Chemical fingerprints of the re-extracted herbal extracts in 80% methanol were obtained using liquid chromatography - electrospray ionization - mass spectrometry (LC-ESI-MS) analyses (not illustrated). LC-ESI-MS experiments were performed on a Waters Acquity Xevo TQ triple quadrupole mass spectrometer coupled to a binary pump, and an autosampler (Waters, Australia). Separation was achieved using a Waters Acquity Xevo TQ triple quadrupole mass spectrometer coupled to an ultra performance liquid chromatography binary pump, a photo-diode-array detector, and an autosampler (Waters, Australia). Separation was achieved on Acquity UPLC BEH C-18 column (150 mm × 2.1 mm, 1.7 μm) attached with a Vanguard™ BEH C18 (1.7 μm) at a mobile phase flow rate of 0.3 ml/min, operating at room temperature. The mobile phase consisted of 0.1% aqueous formic acid (A) and acetonitrile (B). The gradient elution was used with a starting mobile phase composition of 20%, increasing to 100% B for 16 min. Mass spectra were acquired in both positive and negative ionization mode using an ESI source with the mass recorded in the range of m/z 50 – 1000.

### Statistical analysis

One-way ANOVA tests were carried out with GraphPad Prism (version 5.03). Data from triplicate cultures in 2–3 separate experiments are presented as mean ± SEM. Data comparison between drug treated and untreated groups were made by Dunnett post tests. A *p* value < 0.05 was considered statistically significant and denoted by asterisks. The relationships between antioxidant capacity and cytotoxicity data were assessed by calculation of Pearson’s correlation coefficients.

## Results and discussion

### Some herbal extracts exhibit toxic effects in cultured primary cortical neurons

**Figure 1:**
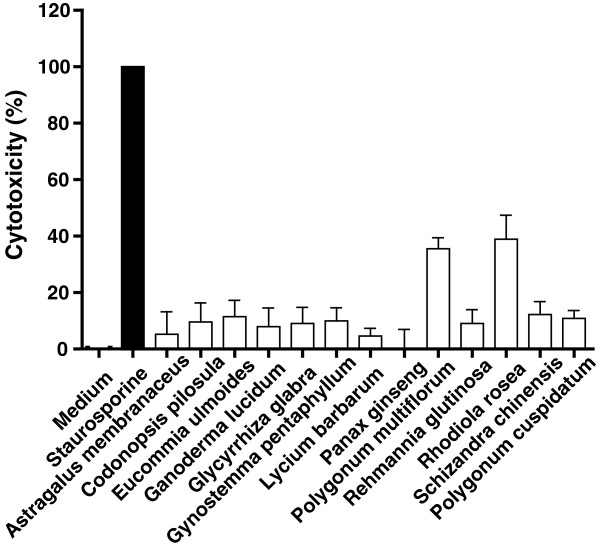
**Cytotoxic effects of herbal extracts in cultured primary mouse cortical neurons.** The neuronal cells were exposed to 100 μg/ml of the herbal extracts for 24 h. Culture media (neurobasal medium supplemented with 2% B-27) containing vehicle only were used as a negative control (defined as 0%) and media containing staurosporine were used as positive control (defined as 100%).

First, we determined whether the herbal extracts (100 μg/ml for 24 hours) exerted toxic effects in cultured mouse cortical neurons. The results demonstrated that most of the herbal extracts showed negligible cytotoxic effects when compared to the positive control of staurosporine-induced cytotoxicity (defined as 100%). However, *Polygonum multiflorum* and *Rhodiola rosea* showed approximately 40% of cytotoxic effects compared to staurosporine-induced cytotoxicity at the highest concentration used in this study (Figure 1). This finding provided experimental evidence that this group of traditional herbal medicines might be more toxic than traditionally claimed. Further studies are warranted to elucidate potentially toxic component(s) in the extracts, followed by animal studies to assess any *in vivo* toxicity. At this time, however, caution should be exerted before any of the herbal medicines that exhibited neurotoxic effects *in vitro* can be recommended for safe long-term use as in humans.

### Neuroprotective effects of herbal extracts

**Figure 2:**
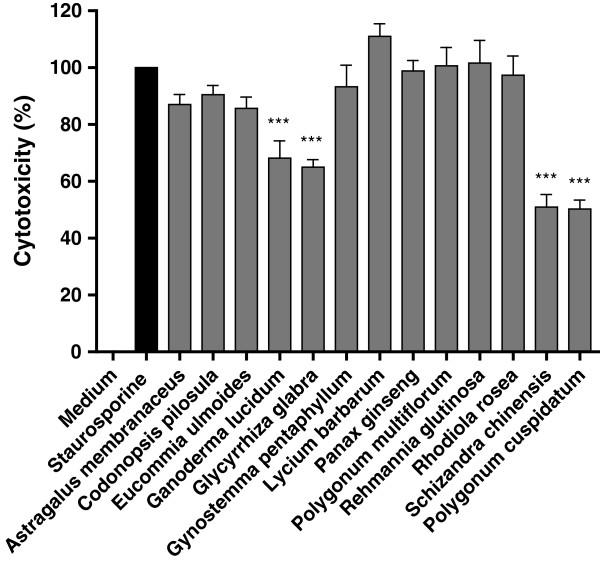
**Neuroprotective effects of the herbal extracts.** The relative cytotoxicity of neuronal cells was measured for the cell cultures (n = 3) co-treated with 100 μg/ml of the herbal extracts and 500 nM staurosporine for 24 h. * *p* value < 0.05; ** *p* value < 0.01, and *** *p* value < 0.001 compared with the positive control.

**Figure 3:**
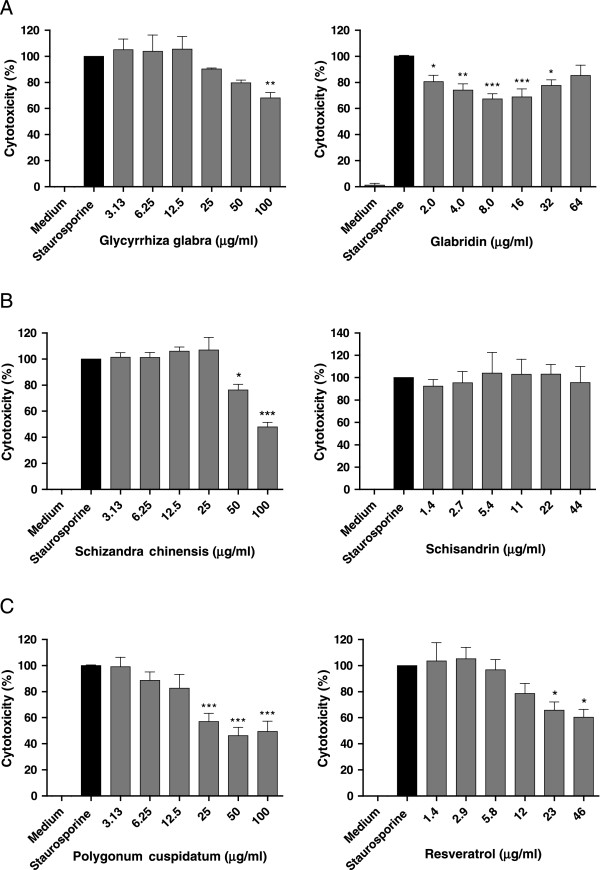
**Dose-dependence of neuroprotective effects of selected herbal extracts and purified compounds.** The herbal extracts, *Glycyrrhiza glabra***(A, left panel)**, *Schizandra chinensis* is **(B, left panel)** and *Polygonum cuspidatum***(C, left panel)**, were used in the concentration range of 3 – 100 μg/ml. The pure compounds, glabridin **(A, right panel)**, schisandrin **(B, right panel)** and resveratrol **(C, right panel)** were used in the range of 1 – 65 μg/ml. * *p* value < 0.05; ** *p* value < 0.01, and *** *p* value < 0.001 compared with the positive control.

The 13 herbal extracts were subsequently investigated for neuroprotective effects by co-treatment of the cells during staurosporine exposure. *Ganoderma lucidum*, *Glycyrrhiza glabra*, *Schizandra chinensis* or *Polygonum cuspidatum*, significantly reduced staurosporine-induced apoptotic cell death by 32 ± 6.1%, 35 ± 2.7%, 49 ± 4.4% and 50 ± 3.2%, respectively (Figure 2). The dose response of the neuroprotective effects of *G. glabra*, *S. chinensis* and *P. cuspidatum* were examined using concentrations of 3 – 100 μg/ml (Figure 3). Interestingly, *P. cuspidatum* exhibited no protective effects in hydrogen peroxide treated U373 astroglial cells [15].

Next, we examined whether known active ingredients in the herbal extracts, *G. glabra*, *S. chinensis* and *P. cuspidatum*, accounted for or at least contributed to the observed neuroprotective activity of the extracts. We examined glabridin (Figure 3A, right) from *G. glabra* (Figure 3A, left), schisandrin (Figure 3B, right) from *S. chinensis* (Figure 3B, left) and resveratrol (Figure 3C, right) from *P. cuspidatum* (Figure 3C, left) for their neuroprotective activity using the same procedure as for herbal extracts, but over the concentration range of 1 – 65 μg/ml. The maximal level of neuroprotective activity of glabridin was 30% inhibition of staurosporine-induced cell death at 8–16 μg/ml. Resveratrol exhibited 40% inhibition at 23–46 μg/ml. The higher concentrations resulted in less activity because the beneficial activity appeared to be offset by toxic effects of the compounds. In the example of glabridin, high concentrations (> 16 μg/ml) resulted in cytotoxic effects in a dose-dependent manner. Schisandrin did not exhibit neuroprotective activity (Figure 3).

*P. cuspidatum* is widely used as a traditional medicinal herb in Asia. An increasing body of research has demonstrated that the extracts from the root of this herb possess antioxidant activity, anti-proliferative effects on cancer cells, wound healing and, in accordance with our data, neuroprotective activities [19-22]. A large number of chemical components have been isolated from this herb including anthraquinones, stilbenes, flavonoids, lignin and their derivatives [23]. In Kim et al.’s study, one of the fractions from the *P. cuspidatum* extract, containing the highest stilbene and anthraquinones, showed the strongest neuroprotective effects in both a non-celluar antioxidant assay and in a rat model of transient cerebral ischemia [22]. Our data showed that a standardized *P. cuspidatum* extract containing 50% resveratrol (confirmed by HPLC/MS analysis, not illustrated) exhibited both potent anti-oxidant capacity and neuroprotective effects. We further found that resveratrol itself was neuroprotective suggesting that neuroprotective effects of the *P. cuspidatum* extract in our experiments was predominantly due to its content of resveratrol.

Resveratrol has stimulated considerable research interest due to its pharmacological effects including antioxidant, anti-aging, anti-inflammatory and anti-neoplastic activities, and immune regulation. Interestingly, in our study, the herbal extract with molar equivalent amount of resveratrol showed slightly higher neuroprotective activity than pure resveratrol, suggesting multiple components apart from resveratrol in the extract may be involved in and additive to the positive effects. Although *P. multiflorum* is a close relative of *P. cuspidatum* in the Polygonaceae family, the former did not exhibit neuroprotective effects in our experiments, but proved to be toxic to neuronal cells. Instead of resveratrol, which was not detected in the extract (data not shown), *P. multiflorum* contains a resveratrol derivative, 2,3,5,4′-tetrahydroxystilbene 2-O-β-D-glucoside (THSG) as the main bioactive component [24]. Compared to *P. cuspidatum* extract, relatively, *P. multiflorum* extract had slightly lower, but still strong antioxidant capacity determined by the non-cellular assays. Our data suggests that the glycation of resveratrol (to form stilbene glucoside) may prevent *P. multiflorum* from having neuroprotective effects in staurosporine-induced apoptosis. In contrast to its lack of neuroprotective activity, *P. multiflorum* exhibited potent cytoprotective activity in hydrogen peroxide treated astroglial cells [15].

*S. chinensis* also exhibited significant neuroprotective activity comparable with *P. cuspidatum*. Since the major constituents of *S. chinensis* are lignans, among which, schisandrin is present at the highest concentration [25], we tested various concentrations of pure schisandrin but could not detect any neuroprotective effects. Further work will be required to identify the neuroprotective components of this extract. The relatively small neuroprotective effect of *G. glabra* was compared with glabridin, a major flavonoid of *G. glabra*. Glabridin showed a significant neuroprotective effect in a dose-dependent manner, but in a narrow range up to 10 μg/ml, confirming the previously published neuroprotective effect of glabridin [26].

### Antioxidant activity of herbal extracts and the correlation with their neuroprotective activities

We determined the antioxidant capacity of the extracts using three non-cellular assays measuring the total phenol content (FCR assay), 2,2-diphenyl-1-picrylhydrazyl (DPPH) radical scavenging capacity and oxygen radical absorbance capacity (ORAC). The ranking of their antioxidant activity and phenolic content showed a similar pattern in the three assays [15]. The three herbal extracts with the highest antioxidant activity were *Rhodiola rosea* > *P. cuspidatum* > *P. multiflorum* (DPPH) and *P. cuspidatum* > *P. multiflorum* > *R. rosea* (ORAC); and with the highest phenolic content were in *P. cuspidatum* > *R. rosea* > *P. multiflorum* (FCR).

**Figure 4:**
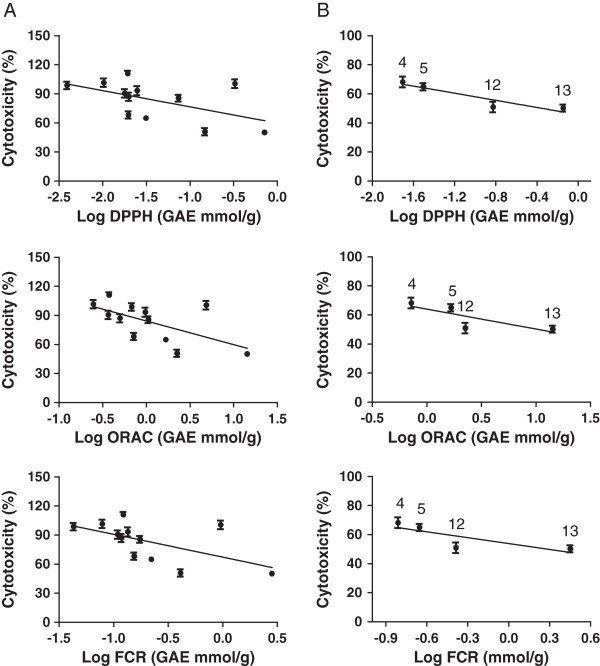
**Correlation analysis between the neuroprotective activity and antioxidant capacity of the herbal extracts.** The neuroprotective activity of the herbal extracts was normalized to the cytotoxicity of staurosporine (100%). The antioxidant activities were determined by non-cellular DPPH, ORAC and FCR assays. Gallic acid equivalence (GAE) was used for relative quantification of the antioxidant activity. The correlation curves were plotted and the correlation level was analyzed for all 13 herbal extracts **(A)** and for selected bioactive herbal extracts **(B)**, *G. lucidum* (4), *G. glabra* (5), *S. chinensis* (12), and *P. cuspidatum* (13), using GraphPad Prism software.

Comparison of the data from the antioxidant capacity assays with those from the staurosporine assay suggested that they appeared to be correlated. Correlation analysis confirmed the strong correlation with the correlation co-efficient (*r*) equal to 0.93 (DPPH), 0.80 (ORAC) and 0.81 (FCR) respectively (Figure 4). The data suggest that the neuroprotective activity of these herbal extracts was correlated with their antioxidant capacity and phenolic content determined in non-cellular assays. Interestingly, however, the *P. multiflorum* and *R. rosea* extracts proved to be the exception as they exhibited a high level of antioxidant capacity in all three assays, but did not exhibit neuroprotective effects in the cell-based assay. These results suggest that high antioxidant activity alone is not a predictor of cytoprotective activity and the mechanistic contribution of antioxidant effects of plant secondary metabolites needs to be established on a case-by-case basis. It is possible that antioxidant effects of plant-derived compounds are merely epiphenomena; easily assayed but not necessarily biologically relevant.

## Conclusion

The results from this study demonstrate that *G. lucidum*, *G. glabra*, *S. chinensis* and *P. cuspidatum* significantly protected primary cortical neurons from staurosporine induced cell death. The neuroprotective effects of the *P. cuspidatum* extract appeared to be predominantly due to its major ingredient, resveratrol, but were more effective based on equivalence of resveratrol concentrations. The neuroprotective activity of these herbal extracts was correlated with their antioxidant capacity. Interestingly, some extracts exhibited selective protection in neurons but not glia (*P. cuspidatum*) or glia but less so in neurons (*P. multiflorum*). Together, the data appear to support the traditional practice of combining multiple herbal medicines for the treatment of complex disorders [15].

Our findings provide experimental evidence that justify future *in vivo* studies to lay the ground for or finally refute the scientific and rational use of this category of herbal medicines to promote healthy aging. At present, however, caution is warranted as some herbal medicines may in fact exhibit neurotoxic effects.

## Competing interests

The authors declare that they have no competing interests.

## Authors’ contributions

BS carried out the cell culture experiments, participated in data analysis and manuscript writing. JT carried out the anti-oxidant assays and phytochemical analysis. RH participated in data analysis and manuscript writing. SG participated in the design of the study, phytochemical analysis, data analysis and manuscript writing. NJS conceived of the study, and participated in its design and coordination and wrote the initial and final draft the manuscript. All authors read and approved the final manuscript.

## Pre-publication history

The pre-publication history for this paper can be accessed here:

http://www.biomedcentral.com/1472-6882/13/373/prepub

## Supplementary Material

Additional file 1: Table S1 Traditional Chinese herbal medicines used in this study, their extraction and major phytochemical constituents. Click here for file
