# Supplementary material for: An in vitro study of neuroprotective properties of traditional Chinese herbal medicines thought to promote healthy ageing and longevity
Source: BMC Complement Altern Med. 2013 Dec 27;13:373. doi: 10.1186/1472-6882-13-373 (PMC3880008; doi:10.1186/1472-6882-13-373)
Supplement: Additional file 1: Table S1 — Traditional Chinese herbal medicines used in this study, their extraction and major phytochemical constituents. [file 1472-6882-13-373-S1.docx]

**Table S1. Traditional Chinese herbal medicines used in this study, their extraction and major phytochemical constituents**

| **No.** | **Medicinal Name** | **Scientific Name** | **Chinese Name (Pinyin)** | **Extraction (ethanol: water; %)** | **Phytochemical constituents** |
| --- | --- | --- | --- | --- | --- |
| 1 | Radix astragali | *Astragalus membranaceus* | Huangqi | 0:100 | Cycloartane triterpene glycosides (astragalasides I-VII); flavonoids [[1](#_ENREF_1)] |
| 2 | Radix codonopsis | *Codonopsis pilosula* | Dangshen | 50:50 | Phenylpropanoids and lignans (tangshenosides, syringin,syringalehyde, coniferyl alcohol, pinoresinol) [[2](#_ENREF_2)] |
| 3 | Cortex eucommiae | *Eucommia ulmoides* | Duzhong | 70:30 | Lignans (derivatives of pinoresinol, syringaresinol, and olivil); iridoid glycosides and phenylpropanoids [[3](#_ENREF_3)] |
| 4 | Ganoderma | *Ganoderma lucidum* | Lingzhi | 30:70 | Lanostane triterpenoids (ganoderic acids) [[4](#_ENREF_4)] |
| 5 | Radix Glycyrrhizae | *Glycyrrhiza glabra* | Ganzao | root powder | Triterpene glycosides (glycyrrhizin); flavonoids (liquiritin); chalcones (isoliquiritin); isoflavones (glabridin) [[5](#_ENREF_5)] |
| 6 | Gynostemma pentaphyllum | *Gynostemma pentaphyllum* | Jiaogulan | 70:30 | Triterpene (dammarane) glycosides (gypenosides) [[6](#_ENREF_6)] |
| 7 | Fructus lycii | *Lycium barbarum* | Gouqi | 65:35 | Polysaccharides; carotenoids (zeaxanthin mono and di-palmitate); phenylcarboxylic acids; flavonoids [[7](#_ENREF_7)] |
| 8 | Radix ginseng | *Panax ginseng* | Renshen | 50:50 | Triterpene glycosides (ginsenosides: dammarane and oleanane skeletons) [[8](#_ENREF_8)] |
| 9 | Caulis polygoni multiflori | *Polygonum multiflorum* | Heshouwu | 80:20 | Stilbenes; hydroxyanthra-quinones; flavonoids [[9](#_ENREF_9)] |
| 10 | Radix rehmanniae | *Rehmannia glutinosa* | Dihuang | 60:40 | Iridoid glycosides (catalpol, aucubin) and phenolics (forsythiaside) [[10](#_ENREF_10)] |
| 11 | Radix rhodiolae | *Rhodiola rosea* | Hongjingtian | 60:40 | Phenylpropanoids (rasavin, rosin, rasarin); Phenylethanoids (salidroside, tyrosol); Flavonoids (rodiolin, rodionin, tricin) [[11](#_ENREF_11)] |
| 12 | Fructus schisandrae chinensis | *Schizandra chinesis* | Wuweizi | 95:5 | Lignans (gomisin, schzandrin and related) [[12](#_ENREF_12)] |
| 13 | Rhizoma polygoni cuspidati | *Polygonum cuspidatum* | Huzhang | confidential process | Stilbenes (trans-resveratrol, polydatin); hydroxyanthraquinones (anthraglycoside B, emodin, physcion) [[13](#_ENREF_13)] |

1. Ma XQ, Shi Q, Duan JA, Dong TT, Tsim KW: **Chemical analysis of Radix Astragali (Huangqi) in China: a comparison with its adulterants and seasonal variations**. *Journal of agricultural and food chemistry* 2002, **50**(17):4861-4866.

2. Qiao CF, He ZD, Han QB, Xu HX, Jiang RW, Li SL, Zhang YB, But PPH, Shaw PC: **The Use of Lobetyolin and HPLC-UV Fingerprints for Quality Assessment of Radix Codonopsis**. *Journal of Food and Drug Analysis* 2001, **15**(3):258-264.

3. Deyama T, Nishibe S, Nakazawa Y: **Constituents and pharmacological effects of Eucommia and Siberian ginseng**. *Acta pharmacologica Sinica* 2001, **22**(12):1057-1070.

4. Gao JJ, Nakamura N, Min BS, Hirakawa A, Zuo F, Hattori M: **Quantitative determination of bitter principles in specimens of Ganoderma lucidum using high-performance liquid chromatography and its application to the evaluation of ganoderma products**. *Chemical & pharmaceutical bulletin* 2004, **52**(6):688-695.

5. Chin YW, Jung HA, Liu Y, Su BN, Castoro JA, Keller WJ, Pereira MA, Kinghorn AD: **Anti-oxidant constituents of the roots and stolons of licorice (Glycyrrhiza glabra)**. *Journal of agricultural and food chemistry* 2007, **55**(12):4691-4697.

6. Razmovski-Naumovski V, Huang T-W, Tran V, Li G, Duke C, Roufogalis B: **Chemistry and Pharmacology of Gynostemma pentaphyllum**. *Phytochem Rev* 2005, **4**(2-3):197-219.

7. Amagase H, Farnsworth NR: **A review of botanical characteristics, phytochemistry, clinical relevance in efficacy and safety of Lycium barbarum fruit (Goji)**. *Food Research International* 2011, **44**(7):1702-1717.

8. Christensen LP: **Chapter 1 Ginsenosides: Chemistry, Biosynthesis, Analysis, and Potential Health Effects**. In: *Advances in Food and Nutrition Research.* Edited by Steve LT, vol. Volume 55: Academic Press; 2008: 1-99.

9. Xu YL, Dong Q, Hu FZ: **Simultaneous quantitative determination of eight active components in**

**Polygonum multiflorum Thunb by RP-HPLC**. *Journal of Chinese Pharmaceutical Sciences* 2009, **18**(4):358-361

10. Zhang RX, Li MX, Jia ZP: **Rehmannia glutinosa: review of botany, chemistry and pharmacology**. *Journal of ethnopharmacology* 2008, **117**(2):199-214.

11. Panossian A, Wikman G, Sarris J: **Rosenroot (Rhodiola rosea): traditional use, chemical composition, pharmacology and clinical efficacy**. *Phytomedicine : international journal of phytotherapy and phytopharmacology* 2010, **17**(7):481-493.

12. Wang M, Wu Q-L, Tadmor Y, Simon James E, Sang S, Ho C-T: **Schisandra chinensis: Chemistry and Analysis**. In: *Oriental Foods and Herbs.* vol. 859: American Chemical Society; 2003: 234-246.

13. Zhang W, Jia Y, Huang Q, Li Q, Bi K: **Simultaneous Determination of Five Major Compounds in Polygonum cuspidatum by HPLC**. *Chroma* 2007, **66**(9-10):685-689.
